# THE LTSS STATE SCORECARD AND MULTISECTOR PLANS FOR AGING

**DOI:** 10.1093/geroni/igad104.0592

**Published:** 2023-12-21

**Authors:** Narda Ipakchi

**Affiliations:** The SCAN Foundation, Long Beach, California, United States

## Abstract

Strong, comprehensive plans are crucial to secure lasting success in state LTSS systems. State Multisector Plans for Aging meet this need and are present and/or under development in more than a dozen states and DC. A Multisector Plan for Aging, a concept led by The SCAN Foundation, is a blueprint that: a) includes planning for 10 or more years; b) is often led by a governor with other executive and legislative leaders; and c) is developed to guide the restructuring of state and local policy, programs, and funding toward aging well in the community. For the first time, the LTSS State Scorecard credits states for having these plans. This portion of the symposium will describe Scorecard findings on Multisector Plans for Aging and address more broadly the important role of these plans to state leadership.

